# Correction: Bis(amidophenolate)-supported pnictoranides: Lewis acid-induced electromerism in a bismuth complex

**DOI:** 10.1039/d6sc90049f

**Published:** 2026-03-16

**Authors:** Simon B. H. Karnbrock, Jan F. Köster, Isabelle Becker, Christopher Golz, Franc Meyer, Martí Gimferrer, Manuel Alcarazo

**Affiliations:** a Institute of Organic and Biomolecular Chemistry, University of Göttingen Tammannstraße 2 37077 Göttingen Germany manuel.alcarazo@chemie.uni-goettingen.de; b Institute of Inorganic Chemistry, University of Göttingen Tammannstraße 4 37077 Göttingen Germany; c Institute of Physical Chemistry, University of Göttingen Tammannstraße 6 37077 Göttingen Germany

## Abstract

Correction for “Bis(amidophenolate)-supported pnictoranides: Lewis acid-induced electromerism in a bismuth complex” by Simon B. H. Karnbrock *et al.*, *Chem. Sci.*, 2025, **16**, 14178–14185, https://doi.org/10.1039/D5SC03374H.

Ref. 5*a* within the original manuscript was given as W.-H. Xu, Y.-B. Huang, W.-W. Zheng, S.-Q. Su, S. Kanegawa, S.-Q. Wu, O. Sato, *Chem. Sci*. 2024, **53**, 2512–2516 which was incorrect. The correct reference is given here as ref. 1.

The Royal Society of Chemistry apologises for these errors and any consequent inconvenience to authors and readers.
